# Supplementary material for: Administration of lower doses of radium-224 to ankylosing spondylitis patients results in no evidence of significant overall detriment
Source: PLoS One. 2020 Apr 30;15(4):e0232597. doi: 10.1371/journal.pone.0232597 (PMC7192484; doi:10.1371/journal.pone.0232597)
Supplement: Attachment — Submitted filename: Responses to the reviewer comments.docx [file pone.0232597.s001.docx]

**Responses to the reviewer comments which were very helpful:**

Reviewer 1:

#1: All the references have been changed to the Plos One style as well as the citations in the text.

#2: On line 88 we added the fact that the females consisted of less than 10% of the patients. Including them would increase the complexity of the analysis.

#3: The analysis using Cox Proportional Hazard Regression does adjust for competing risks.

#4: Lines 221-223 were added to the discussion section which suggests an additional concern with our conclusions.

Reviewer 2:

Material has been added to the discussion. Also the non-treated patients were those attending a hospital that objected to using radium in treatment. A sentence was added to the discussion about this being a concern.

#1: Line 122 was added which explains the follow-up.

#2: Line 119 adds more detail about the variables in the Cox model.

#3: Line 419 begins an explanation for the differences in proportion of deaths (age).

#4: Table 1 is now included. This was a simple error by not having included it.

#5: All confidence intervals now clearly state that they are 95% intervals.

#6: HRs are now used through out the text.

#7: This is corrected. Line 153.

#8: This has been corrected.

#9: It is now stated that for the analysis it was necessary to assume that the population were identical. We continue to say they actually are not (different average ages) and different hospitals etc.

#10: I believe Fig. 6 is meant here and not Fig. 2. We prefer to present a figure instead of a table of number of cases especially when the various groups have different aver ages. If necessary we could simply remove the figure from the manuscript.
